# Plant biomass as potential economic commodities for agricultural purposes

**DOI:** 10.3389/fchem.2022.806772

**Published:** 2022-09-07

**Authors:** Veronica C. Obuseng, Mohau N. Moshoeshoe, Florence M. Nareetsile, Habauka Kwaambwa, Irene Maina

**Affiliations:** ^1^ Department of Chemistry, University of Botswana, Gaborone, Botswana; ^2^ National University of Lesotho, Roma, Lesotho; ^3^ Department of Natural and Applied Sciences, Namibia University of Science and Technology, Windhoek, Namibia; ^4^ Department of Chemical and Forensic Sciences, Botswana International University of Science and Technology, Palapye, Botswana

**Keywords:** heavy metal, nitrates, nitrites, phosphates, wastewater

## Abstract

The world’s population is growing continually and is projected to reach nine billion by the year 2050. This growth rate requires increased and economically viable food production and an adequate supply of quality water to sustain life. Increased food production and supply of water require adding fertilizers and possible recycling of wastewater, to address the improvement of soils’ nutritional status and potable water shortages, respectively. The objectives of this work were to determine the nutrients in sewage-impacted wastewater, borehole water, agricultural waste, and commercial fertilizer (control) materials, and their heavy metal content was also carried out to determine their suitability for use. In addition, Moringa seed pods and Morula nutshells were investigated as a bioremedial approach for the removal of toxic metals from aqueous samples. An attempt to regenerate sorbent was made since the saturated sorbents that contain the metal ions are not safe for disposal as they can pollute the environment. Nutrients were analyzed by HPLC, while metals were analyzed using a Varian 220FS Atomic Absorption Spectrometer operated with air/acetylene. Nonedible agricultural materials were found to contain appreciable amounts of plant nutrients such as nitrates (NO_3_^-^), nitrites (NO_2_^-^), and phosphates (PO_4_^3-^) as well as metal ions such as magnesium, copper, and zinc, which are beneficial for plant growth. Results obtained from analysis of sewage water effluent showed that heavy metal and nutrient concentrations decreased in the treatment stage. The utilization of *Moringa oleifera* seed pods for metal removal from wastewater is viable and would reduce costs for waste disposal and can offer alternatives to conventional methods for the removal of unwanted or toxic species from the environment. It showed potential for removing selected metal ions such as Pb, Cd, Cu, Fe, and Zn from polluted water. This organically treated wastewater is environmentally friendly and may be used for applications which do not require potable water, such as irrigating golf courses, lawns, and crops, or for industrial purposes, if proper measures are taken to ensure its quality.

## 1 Introduction

Plants serve as the main source of food for humanity. These plants, in turn, need nutrients for their growth and other life processes, which they source mainly from soil. A fertile soil should contain proper, balanced levels of the different nutrients. In many cases, these nutrients often fall short of the optimum levels and must be supplemented by the use of fertilizers. Inorganic fertilizers such as ammonium nitrate, potassium nitrate, and urea as well as green manures such as organic compost (Melaj and Daraio, 2013; Babrauskas, 2016), and wastewater treatment sludge (Zorpas et al., 2000) have been utilized for this purpose. Some of these soil amendment strategies introduce components that may be harmful to the environment, leading to environmental concerns such as nitrate water pollution (Chaudhry and Malik, 2017; Singh and Craswell, 2021), the introduction of potentially toxic trace metals (Chaudhry and Malik, 2017). The presence of NO_3_^−^ in food and drinking water is a health concern because the microbial reduction process in food products and saliva may act as carcinogens (Alonso et al., 2020) and cause other related illnesses.

Biosorption has gained important credibility during recent years as a low-cost, readily available, and efficient treatment technology for the high-capacity removal of heavy metals from wastewaters (Bashir et al., 2019; Redha, 2020). For example, acid-treated soybean hulls were used to effectively remove metal ions (Cd, Ni, Cu, Pb, and Zn) from industrial and municipal wastewater. Orange peels have also been used for the removal and recovery of Ni^2+^ ions from electroplating water. The effectiveness of metal removal was dependent on operational conditions such as temperature, pH, initial concentration, and sorbent dose. Other adsorbents which have been used include *Moringa oleifera* seed pods and *Sclerocarya birrea* (Morula) nutshells.

*Moringa oleifera* (MO) and *Sclerocarya birrea* (Morula) are essential for food security, health and nutrition, and economic welfare of rural communities in the developing world (Mojeremane and Tshwenyane, 2004). The Morula tree is a multipurpose tree with highly nutritive fruits which can be consumed fresh or commercially processed. Its fruits are used in the preparation of juices, jams, jellies, and alcoholic beverages as they contain high amounts of vitamin C (Mojeremane and Tshwenyane, 2004; Ojewole et al., 2010; Moatshe et al., 2011; Maina et al., 2016). The seed biomass has been used for water treatment for generations in several countries by local communities (Nwagbara et al., 2022).

Nutrients (NO_3_^−^, NO_2_^−^, and PO_4_^3-^) as well as heavy metals (Pb, Cd, Cu, Fe, and Zn) are commonly monitored for environmental protection purposes in natural and wastewaters as well as in agricultural and food samples (Lockhart et al., 2013; Yun et al., 2018; Mehri et al., 2021). Although techniques such as electrophoresis, electroanalytical methods, GC, IEC, and HPLC have been successfully applied in the determination of nitrates, nitrites, and phosphates, very few applications carried out simultaneous analysis of the three analytes. HPLC equipped with an ultraviolet (UV) detector in the analysis of NO_3_^−^, NO_2_^−^, and PO_4_^3-^ has proven to afford high efficiency, precision, accuracy, sensitivity, and specificity and reduced interference (Moshoeshoe and Obuseng, 2018).

When analyzing solid samples using HPLC, several sample preparation procedures exist which are meant to make the sample compatible with the technique of analysis and reduce the effects of matrix components, thus reducing interferences during analysis. The sample preparation procedures used to extract NO_3_^−^, NO_2_^−^, and PO_4_^3-^ in liquid matrices may include dissolution with hot water (Brkić, et al., 2017; Ferreira and Silva, 2008) and homogenization (Nam et al., 2008). The use of extraction procedures such as the Bremner method (López Pasquali et al., 2010), adsorption on activated, carbon and the alkaline extraction methods (Prasad and Chetty, 2008) may also be necessary. For solid matrices, pretreatment procedures may include drying (Elrashidi et al., 2003, Ferreira and Silva, 2008) or freeze-dry techniques (Prasad and Chetty, 2008), mechanical shaking (Turrión et al., 1999), ultrasonic extraction, and/or blending (Butt et al., 2001). Use of extraction solvents such as hot water (Ferreira and Silva, 2008) or cold water (Burns, 2000) with or without salts or pH adjustment, clean-up techniques such as activated charcoal (Ferreira and Silva, 2008), deproteinization agents, for e.g., Carrez solution (Bashiry et al., 2016; Ferreira and Silva, 2008), or a solid phase extraction column (Jobgen et al., 2007) may also be needed. Although these procedures have been developed to improve the selectivity, specificity, and detection of the analytes of interest, the existing wide range of procedures may have been the source of variations in analytical results in the literature. Based on the comparison of their performances in the literature, five extraction methods were selected and examined. The selection of these extraction methods was based on 1) ability of the method to simultaneously extract nitrate (NO_3_^−)^, nitrite (NO_2_^−^), and phosphate (PO_4_^3-^), without oxidizing the nitrite to nitrate; 2) specificity of the extraction technique was also considered, with preference given to methods which can extract only the analytes of interest; 3) “soft” extraction methods (i.e., those methods which extract only the readily available analyte) were also favored over those methods which extract analyte; 4) use of chemical reagents that have safety concerns (all methods which use such reagents were disregarded); and 5) sample throughput, whereby procedures which are simple were preferred over those which are complicated. This also reduces the possibility of artefact production, possible sample contamination, and/or analyte loss.

Extraction using boiling water also satisfies all the aforementioned requirements as water is expected to be able to dissolve all ionic compounds. Moreover, water is non-toxic, and fewer steps are used in the extraction. The alkaline extraction method and the modified alkaline extraction method employ the use of NaOH as an extracting agent. ZnSO_4_ is later used as a precipitating agent to remove most unrequired ions from solution. Both extraction procedures also satisfy all the aforementioned requirements as they use non-toxic and specific reagents, which are also capable of the simultaneous extraction of the analytes of interest. Boiling water, alkaline extraction and modified alkaline extraction methods, and oxalate extraction and AOAC extraction methods were selected and compared for extraction of NO_3_^−^, NO_2_^−^, and PO_4_^3-^ in a commercial NPK fertilizer. This article showcases some applications of agricultural waste (non-edible parts) such as *Moringa oleifera* seed pods, maize plant stalks, and Jugo bean husks as potential organic materials for wastewater treatment and soil amendment. The objectives of this work were to determine the nutrients in sewage-impacted wastewater, borehole water, agricultural waste, and commercial fertilizer (control) materials, and their heavy metal content was also carried out to determine their suitability for use. In addition, Moringa seed pods and Morula nutshells were investigated as a bioremedial approach for the removal of toxic metals from aqueous samples. An attempt to regenerate sorbent was made since the saturated sorbents that contain the metal ions are not safe for disposal as they can pollute the environment.

## 2 Materials and methods

### 2.1 Materials

Commercially available reagents and solvents were used without further purification. Activated carbon, oxalic acid (H_2_C_2_O_4_), calcium chloride (CaCl_2_), EDTA (C_10_H_14_N_2_Na_2_O_8_•2H_2_O), and zinc sulfate (ZnSO_4_.7H_2_O) were obtained from Merck (PTY) Ltd. (Modderfontein, South Africa). Orthophosphate, nitrite, and nitrate stock solutions were prepared using potassium dihydrogen phosphate (KH_2_PO_4_), sodium nitrite (NaNO_2_), and sodium nitrate (NaNO_3_), respectively, all of which were purchased from Saarchem (Krugersdorp, South Africa). Potassium thiocyanate (KSCN) and diethylenetriaminepentaacetic acid (DTPA) were also purchased from Saarchem (Krugersdorp, South Africa). Sodium hydroxide (NaOH), potassium chloride (KCl), ammonium oxalate ((NH_4_)_2_C_2_O_4_•H_2_O), ammonium bicarbonate (NH_4_HCO_3_), and ammonium citrate ((NH_4_)_2_HC_6_H_5_O_7_) were all purchased from Rochelle Chemicals, RSA. All reagents used for optimization of extraction methods were of analytical-reagent grade. Distilled, de-ionized water (DDW, 18 MΩ cm^−1^; Milli-Q, Millipore) was used for all sample preparation procedures. All glassware used was soaked in 10% HCl for 24 h and rinsed several times with DDW prior to use.

### 2.2 Extraction of NO_3_^−^, NO_2_^−^, and PO_4_^3-^

Different sample extraction procedures were compared for their efficiency in extracting NO_3_^−^, NO_2_^−^, and PO_4_^3-^ from solid samples using commercial N:P:K fertilizer samples. They were freeze-dried, ground, and sieved through a 2-mm mesh and then processed using the different extraction procedures described in the following paragraphs.

A comparison of the extraction efficiency of the different procedures in extracting NO_3_^−^, NO_2_^−^, and PO_4_^3-^ from liquid samples was carried out using sewage-impacted wastewater samples obtained from the final effluent at the Glen Valley Wastewater Treatment Plant in Gaborone (GVWTP). Liquid samples were filtered first through a Whatman filter paper (No. 1) to remove insoluble materials.

The alkaline extraction procedure described by Prasad and Chetty, (2008) was used without modification. In brief, 10 g of NPK fertilizer was weighed and shaken for 30 min with 70 ml of water in a Labcon shaking water bath. A volume of 12 ml of 2% NaOH was then added, and the water bath temperature was increased to 50°C while shaking. The resulting precipitate was filtered, and then 10 ml of ZnSO_4_ was added to the filtrate, while the temperature of the suspension was maintained at 50°C for further 10 min. The contents were then cooled to room temperature by immersing the flask in tap water. The suspension was then filtered through a 0.45-µm Minisart^®^ Plus syringe membrane filter into a 100-ml volumetric flask, to which was added the internal standard (KSCN) to give a final concentration of 20 mg/L and made up to the mark with water. The resulting solution was analyzed following the HPLC method.

The boiling procedure as described by Chou *et al.* (2003) was employed by weighing 10 g of a sample, adding it to 50 ml of water in an Erlenmeyer flask, and shaking the mixture in a boiling water bath (Labcon shaking water bath) for 20 min. The sample was then shaken up and left on the table until cooled down to room temperature, after which it was filtered through a 0.45-µm Minisart^®^ Plus syringe membrane filter into a 100-ml volumetric flask. The internal standard (KSCN) was added to give a final concentration of 20 mg/L, and the solution was made up to the mark with water. The resulting solution was analyzed using the HPLC method.

The modified alkaline extraction was applied as described by Akyüz and Ata, (2009). A measure of 0.5 M aqueous NaOH was added to 10 g of the sample. The mixture was sonicated at 40°C for 20 min and then filtered using a Whatman filter paper (No. 1). The aqueous phase was collected in a beaker. A measure of 0.5 M ZnSO_4_ was added dropwise to this aqueous phase until the white precipitate of Zn(OH)_2_ no longer appeared on further addition. The aqueous phase containing nitrite and nitrate ions was filtered through a 0.45-µm Minisart^®^ Plus syringe membrane filter into a 50-ml volumetric flask. The internal standard (KSCN) was added to give a final concentration of 20 mg/L, and the solution was made up to the mark with water, followed by HPLC analysis.

The oxalate extraction procedure was adapted from Kleinman & Sharpley, (2007). A measure of 10 g of the sample was shaken in 80 ml of acid oxalate solution (0.1 M (NH_4_)_2_C_2_O_4_•H_2_O+ 0.1 M H_2_C_2_O_4_•2H_2_O) for 4 h in the dark. The resulting extracts were centrifuged for 20 min and then filtered through a 0.45-µm Minisart^®^ Plus syringe membrane filter into a 100-ml volumetric flask. KSCN was added as an internal standard to give a final concentration of 20 mg/L, and the solution was made up to the mark with doubly distilled water (DDW), followed by HPLC analysis.

The AOAC official procedure described by Bartos *et al.* (2014) was used without modification. A measure of 10 g of the sample was added to a 250-ml volumetric flask containing 100 ml ammonium citrate–EDTA solution, previously heated to 65°C. The flask was shaken for 60 min in a water bath at 65°C and 200 rpm. It was then removed from the water bath and left to cool to room temperature. The cool solution was filtered through a 0.45-µm Minisart^®^ Plus syringe membrane filter into a 250-ml volumetric flask. KSCN was added as an internal standard to give a final concentration of 20 mg/L, and the solution was made up to the mark with DDW, followed by HPLC analysis. The obtained concentrations are reported in Table 1.

**TABLE 1:** Average concentrations of NO_2_^−^, NO_3_^−^, and PO_4_^3-^ obtained from NPK fertilizers using different extraction methods.

Analyte concentration (in mg/kg)	Extraction procedure				
	Alkaline extraction	Boiling	Modified alkaline extraction	Oxalate	AOAC
NO_2_^−^ (mg/kg)	41.99 ± 1.84	38.56 ± 1.71	45.65 ± 1.83	41.53 ± 1.82	40.97 ± 1.91
NO_3_^−^ **(**mg/kg)	685.38 ± 22.99	667.52 ± 26.59	701.29 ± 16.91	670.19 ± 26.46	688.56 ± 26.82
PO_4_^3-^ **(**mg/kg)	864.64 ± 8.15	881.89 ± 15.29	992.25 ± 7.37	1,638.56 ± 28.54	1,414.53 ± 15.43

No modifications were made to the described procedures, except for the addition of the internal standard (KSCN). All samples were analyzed within 1 h after sample preparation. All extractions and analyses were carried out in triplicate. The resulting extracts were then analyzed by HPLC. The resultant sample peak area ratios were fitted into the previously calculated calibration equation to yield concentrations, which were then compared to determine the most efficient extraction procedure.

### 2.3 HPLC analysis

An HPLC method for the simultaneous analysis of nitrates, nitrites, and phosphates in several environmental samples developed by Moshoeshoe and Obuseng, (2018) was utilized for all the analyses. HPLC analyses were carried out using an Agilent Technologies HPLC-UV (Agilent Infinity Series 1,260—DAD) equipped with a diode array detector and manual injection using OpenLAB CDS ChemStation software. Detection was carried out at a wavelength of 210 nm, at which absorbance was found to be adequate for all the analytes. Baseline resolution for all the analytes was achieved on a Phenomenex Synergi Polar-RP column (150 × 4.6 mm, 4 μm, 80 Å), using a mobile phase consisting of acetonitrile:acidified water (pH 2.4; 55:45 v/v) in an isocratic elution mode. Optimum results were obtained at a mobile phase flow rate of 0.80 ml/min and a temperature of 30°C. The eluents were degassed manually before being added into the mobile phase reservoir and online after mixing.

### 2.4 Liquid and solid sample collection and nutrient analysis

Borehole water samples were obtained from a local farm in Artesia, Botswana, at two different boreholes using the grab sampling technique. Water samples were stored in dark bottles, which were placed in a cooler box. They were then taken to the laboratory and stored at 4°C until further analysis. Five replicates were filtered through a Whatman filter paper (No. 1), and 0.5 M NaOH was added to each sample, sonicated, and then filtered again using a Whatman filter paper (No. 1). A volume of 0.5 M ZnSO_4_ was added dropwise to the filtrate until precipitation no longer occurred. The mixture was then filtered through a 0.45-µm Minisart^®^ Plus syringe membrane filter into a 50-ml volumetric flask. The internal standard (KSCN) was added to give a final concentration of 20.00 mg/L, and the solution was made up to the mark with water, followed by HPLC analysis.

Wastewater samples were collected from the Glen Valley Wastewater Treatment Plant (GVWTP) in Gaborone, Botswana. Five different samples were collected from the treatment plant at the inlet and final effluent (contact tank). Five replicates were filtered through a Whatman filter paper (No. 1), added to 0.5 M NaOH, sonicated, and then filtered again using a Whatman filter paper (No. 1). A volume of 0.5 M ZnSO_4_ was added dropwise to the filtrate until precipitation no longer occurred. The mixture was then filtered through a 0.45-µm Minisart^®^ Plus syringe membrane filter into a 50-ml volumetric flask. The internal standard (KSCN) was added to give a final concentration of 20 mg/L, and the solution was made up to the mark with water, followed by HPLC analysis (Table 3).

Agricultural waste material was obtained from a farm in Artesia, Botswana, from three different plowing fields after harvest, using the random sampling technique in each field. These included maize stalks and Jugo bean husk samples. *Moringa oleifera* seed pods and Morula nutshells were obtained from areas around Gaborone, Botswana. Grab samples, which were collected from each field, were combined into a single composite sample and freeze-dried. The solid samples were ground using a pestle and mortar. Simultaneous quantitative extraction of NO_3_^−^, NO_2_^−^, and PO_4_^3-^ was obtained when applying the modified alkaline extraction method (Prasad and Chetty, 2008). In brief, ground solid samples were air-dried, after which five replicates were weighed, added to 0.5 M NaOH, sonicated, and then filtered again using a Whatman filter paper (No. 1). A volume of 0.5 M ZnSO_4_ was added dropwise to the filtrate until precipitation no longer occurred. The mixture was then filtered through a 0.45-µm Minisart^®^ Plus syringe membrane filter into a 50-ml volumetric flask. The internal standard (KSCN) was added to give a final concentration of 20 mg/L, and the solution was made up to the mark with water, followed by HPLC determination under optimized conditions as published by Moshoeshoe and Obuseng, (2018).

### 2.5 Determination of heavy metals

#### 2.5.1 Determination of initial metal concentration in water samples

Water samples collected from the sampling points (boreholes and wastewater samples) were acid-digested following a method adopted from the EPA 1999 method 200.2 for wastewater samples. A volume of 50 ml of each water sample from the inlet and effluent points of the GWWTP and borehole was put into 250-ml conical flasks. A volume of 1 ml of 69% HNO_3_ and 0.5 ml of 37% HCl were added to each flask to digest the samples. The mixture was heated until the initial volume was reduced to approximately 0.5 ml. It was then filtered using a 0.45-µm Minisart^®^ Plus syringe membrane filter into a 50-ml volumetric flask, and deionized water was added to the mixture to make up to the mark. The sample was then analyzed with AAS to determine the concentration of six metals (Pb, Fe, Mn, Zn, Cu, and Cd). This was carried out in triplicate.

#### 2.5.2 Determination of initial metal concentration in sorbent materials

A volume of 2 g of each ground sorbent was weighed and then digested with an aqua regia solution of HCl:HNO_3_ in a ratio of 3:1 v/v. Each mixture was put in 100-ml conical flasks and heated at 85 °C until the volume was approximately 1 ml. The resulting volume was filtered using a Whatman No. 1 filter paper into 50-ml volumetric flasks. Deionized water was added to the flasks up to the mark and then analyzed using FAAS, and the initial concentrations are reported in Table 5.

#### 2.5.3 Instrumental analysis

A Varian 220FS Atomic Absorption Spectrometer operated with air/acetylene was used for the determination of six selected metal ions, namely, Pb, Mn, Fe, Zn, Cu, and Cd. Varian hollow cathode lamps for each of the analyzed metals were used as radiation sources. A measure of 1,000 mgL^−1^ stock solutions of metals were used to prepare working standards (in the range of 0.0–5.0 mgL^−1^) in deionized water. The instrument was calibrated manually by aspirating the prepared working standards of the cations of interest one by one into the flame. The samples were then also aspirated manually into the flame for atomization. The instrumental conditions applied were according to the FAAS manual.

### 2.6 Sorption studies

MO seed pods and Morula nutshells were washed with double deionized water and dried in an oven at 105°C for 24 h. The dried samples were ground and then sieved using a 100-µm mesh sieve and stored in glass bottles until further analysis.

Acid treatment of MO seed pods (MSP) and Morula nutshells (MNS**)** was carried out and 15 g of each sorbent was weighed and put in a conical flask, 200 ml of 0.1 M HNO_3_ was added, and the mixture was soaked for 24 h as described in Won *et al.* (2014). The mixture was then filtered using a Whatman No. 1 filter paper, and the sorbent was washed several times with deionized water until the pH of the sorbent was neutral (around pH 7). The sorbent was then dried in an oven at 50°C overnight and then put in a glass bottle for further use. Following the optimized parameters (Maina et al., 2016), the sorbent was used on wastewater samples and the results of the acid-treated and untreated sorbents were compared. In brief, 2 g each of the sorbents (both untreated MSP and MNS) was ground and sieved to a 100 µm particle size. This was added to both wastewater and borehole samples, with pH adjusted to 8 for optimum sorption. A measure of 50 ml of water samples was used, and the mixture was shaken for 120 and 60 min, respectively. The mixture was then filtered using a Whatman No. 1 filter paper, and the filtrate was transferred into a 50-ml volumetric flask and filled with deionized water to the mark, followed by metal ion analysis with AAS. The optimized conditions were applied to both water samples. The analysis was carried out in triplicate, and the concentration was determined by FAAS.

## 3 Results

### 3.1 Nutrient extraction method

The preliminary results obtained when these extraction procedures for NO_3_^−^, NO_2_^−^, and PO_4_^3-^ were applied to commercial NPK fertilizers, with emphasis on the extraction efficiency of a method, including recovery, selectivity, and lack of production of artefacts, are shown in Table 1. In addition, other factors such as the number of steps involved in the method, the duration of the preparation, and ease of the method were also considered.

Simultaneous quantitative extraction of nitrate, nitrite, and phosphate was obtained when applying the modified alkaline extraction method. The higher pH of reagents used in this method prevents the oxidation of NO_2_^−^ to NO_3_^−^ (which occurs in other methods) and is known to lead to sub-standard NO_2_^−^ levels in the results. In addition, the modified alkaline extraction method gave results which closely approximate the amount of plant-available P in soils and fertilizers, together with the estimation of P loss in runoff and subsurface waters. Therefore, the modified alkaline extraction procedure appears to be an effective multi-ion extraction procedure with respect to the extraction of NO_2_^−^, NO_3_^−^, and PO_4_^3-^ and has been used to extract the nutrients in the samples analyzed in this study.

### 3.2 Nutrient in water samples from GVWTP

The wastewater entering the Glen Valley Wastewater Treatment Plant (GVWTP) contains considerable amounts of nitrates, nitrites, and phosphates due to the chemicals used in both households and industries. The purpose of the treatment plant is to reduce the amount of these analytes as the water goes through the different treatment stages.

The amounts of nutrients at the inlet were found to be very high but reduced as the treatment process continued. This can be attributed to the fact that domestic wastewater consists of gray and black wastewater as shown in Table 2. The water obtained from the final effluent tank was found to have 9.11% of the amount of phosphate initially present at the inlet. This translates to a percentage removal of 90.89%. Similar trends were observed for nitrites and nitrates, with a percentage removal of 97.59% and 87.99%, respectively (Table 2).

**TABLE 2:** Efficiency of the GVWTP on nutrient removal and amounts to maximum limits set by WUC (n = 5).

Holding tank	NO_2_^−^ (mg/L)		NO_3_^−^ (mg/L)		PO_4_^3-^ (mg/L)	
	Obtained amount	WUC limit	Obtained amount	WUC limit	Obtained amount	WUC limit
Inlet	1,409.06 ± 63.41	200	173.75 ± 6.17	100	63.34 ± 1.79	30
Final effluent (contact tank)	33.87 ± 0.76	1.5	20.87 ± 0.71	10	5.77 ± 0.28	1.5
Removal efficiency (9%)	97.59	--	87.99	--	90.89	--

Although the percentage removal of these nutrients is high, the amounts of the analytes in the effluent were still above the quality control limits set by the WUC, Botswana, as shown in Table 2. The effluent is, however, environmentally friendly with regards to the analytes of interest and may be used for applications which do not require potable water (such as irrigational or industrial purposes). Some of the water is used to irrigate golf courses, lawns, and crops grown under the Glen Valley Irrigation Project. The concentration levels of the nutrients from the current study contrast with the results obtained by Nkegbe et al. (2005), who found the effluents to be below the maximum allowable effluent levels of <1 mg/L for PO_4_^3-^ and <5 mg/L for NO_3_^−^. The results could indicate the state of the plant during the sampling period, whereby it may not have been functioning accordingly in the removal of the analytes of interest from wastewater. This water, however, undergoes further natural treatment as the effluent is released to maturation ponds and then discharged into the Notwane River. In this study, the presence/absence of pathogenic organisms has not been tested, so the water cannot be recommended for drinking purposes**.**

### 3.3 NO_2_^−^, NO_3_^−^, and PO_4_^3-^ in borehole water

The results obtained from the analysis of the borehole water samples by HPLC are shown in Table 3.

**TABLE 3:** Concentrations of NO_2_^−^, NO_3_^−^, and PO_4_^3−^ in borehole water on a farm (n = 5).

Analyte	Concentration (mg/L)	
	Borehole 1	Borehole 2
NO_2_^−^	80.58 ± 6.86	ND
NO_3_^−^	108.41 ± 12.59	1.22 ± 0.26
PO_4_^3-^	0.23 ± 0.14	1.98 ± 0.87

Groundwater purity depends on various factors, such as the geological conditions of the soil through which the groundwater flows, and some anthropogenic activities (EPA, 2012). The amount of nitrate and nitrite which was found in borehole 1 exceeds 50 mg/L nitrate and 3 mg/L nitrite limits set by the World Health Organization (WHO, 2017). The high concentrations of nitrate and nitrite in this borehole water could be due to the application of inorganic or organic fertilizer and the excretory materials from farm animals as well as wild animals (WHO, 2011). These may contribute to the adulteration of groundwater with various contaminants.

In general, water obtained from borehole 2 contained lower concentration levels of NO_2_^−^, NO_3_^−^, and PO_4_^3-^ that were below the maximum contaminant limits (WHO, 2011), therefore could be safe for human consumption.

### 3.4 NO_3_^−^, NO_2_^−^, and PO_4_^3−^ in fertilizers and agricultural waste products

The obtained amounts of NO_3_^−^, NO_2_^−^, and PO_4_^3-^ in solid agricultural waste samples are shown in Table 4.

**TABLE 4:** Average concentrations of NO_3_^−^, NO_2_^−^, and PO_4_^3-^ in different agricultural waste and soil samples. NPK fertilizer was included as quality standard.

Sample	[NO_2_^−^] (in mg/kg)	[NO_3_^−^] (in mg/kg)	[PO_4_^3-^] (in mg/kg)
Maize stalks	14.45 ± 0.63	98.59 ± 4.02	18.05 ± 0.46
Jugo bean husks	3.92 ± 0.19	254.61 ± 10.93	19.66 ± 0.50
Moringa seed pods	5.35 ± 0.21	165.56 ± 7.06	27.40 ± 0.41
NPK fertilizer (quality standard)	45.65 ± 1.83	701.29 ± 16.91	864.64 ± 8.15

Generally, the nitrite content in the parts was about a tenth of what was in the fertilizer. The lowest amounts of both NO_2_^−^ and NO_3_^−^ were found in Jugo bean husks. In general, the amount of NO_2_^−^ in the samples followed the trend: maize stalks > Moringa seed pods > Jugo bean husks.

The trend observed for NO_3_^−^ in the samples was different from that realized for NO_2_^−^. The amount of NO_3_^−^ in the samples was according to the following trend: Jugo bean husks > Moringa seed pods > maize stalks. Jugo beans have the highest amount of NO_3_^−^ due to being leguminous plants, thus having the highest capacity for nitrogen fixation.

The observed trend for PO_4_^3-^ in the samples was as follows: Jugo bean husks > maize stalks > Moringa seed pods. Phosphates, nitrates, and nitrites were found to be present in agricultural waste samples, albeit at very small concentrations as compared to their concentrations in field soil and fertilizers. The replowing into the soils of these degradable materials could be utilized as fertilizers to improve agricultural yields. Although the samples were found to contain lesser amounts of nutrients than the NPK-fertilizer, if these agricultural waste samples were to be used, the fertility of the arable land could be maintained as most of the nutrients would be returned to the soil.

### 3.5 MSP- and MNS-selected metal ion composition

Table 5 shows the selected metal ion composition in the sorbents, MNS and MSP. Cadmium was not detected in both sorbents, while the other elements were ≤0.12 mg/g. All the analyzed metals had concentrations within the maximum permissible limits for edible plants set by FAO/WHO (2017).

**TABLE 5:** Concentration of selected metals in MSP and MNS biomass.

Sorbent	Metal concentration (mg/g)					
	Pb	Cu	Cd	Fe	Zn	Mn
Maximum permissible limit in edible plants	0.3	3	0.2	20	27.4	2
MSP	0.0175 ± 0.002	0.0120 ± 0.009	BDL	0.1114 ± 0.026	0.0358 ± 0.015	0.0470 ± 0.007
MNS	0.015 ± 0.004	0.0118 ± 0.005	BDL	0.0875 ± 0.031	0.0259 ± 0.033	0.0285 ± 0.007

BDL: below detectable limit.

**FIGURE 1:**
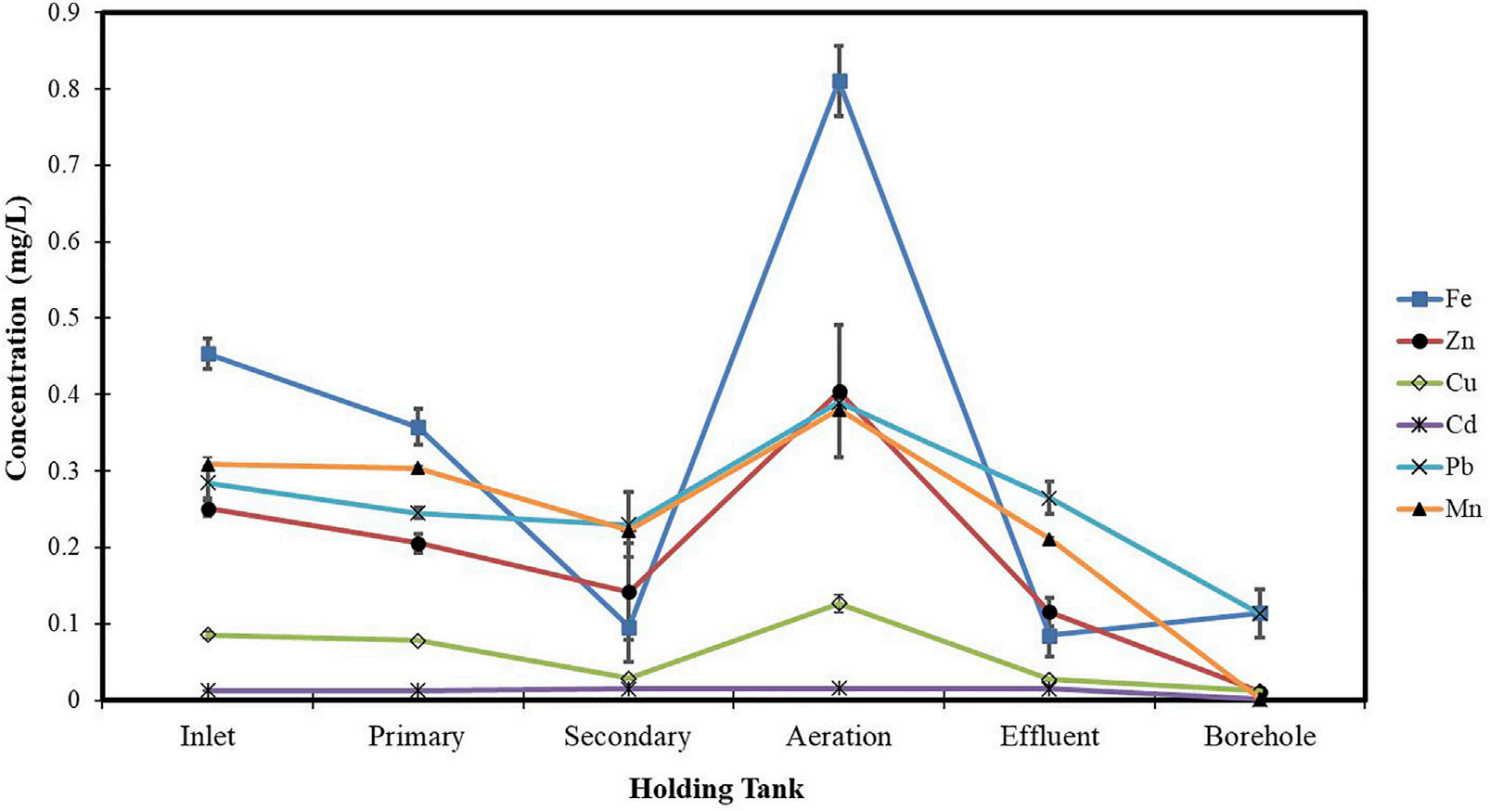
Concentration profile of selected heavy metals along the different holding tanks in the GVWTP.

Figure 1 shows the concentration profile of selected heavy metals along the different holding tanks in the Glen Valley Wastewater Treatment Plant (GVWTP). In general, there was a significant decrease in all the heavy metals throughout the treatment process.

Further work was carried out to determine how effective the MNS and MSP can remove metal ions in the wastewater.

The percentage removal when MNS and MSP were used is given in Table 6. When using MSP, Zn desorbed from MSP into the wastewater, and hence removal of Zn by MSP was impossible. This could be attributed to the saturation of binding sites for Zn in the sorbents.

**TABLE 6:** Percentage removal of selected metals using MNS and MSP.

Sorbent	Water sample holding tank	Percentage removal ± standard deviation of heavy metal				
		Mn	Cu	Fe	Pb	Cd
MNS	Inlet	64.7 ± 1.24	67.3 ± 3.59	84.4 ± 1.94	67.4 ± 3.63	31.2 ± 0.38
	Effluent	56.8 ± 1.68	60.4 ± 2.19	64.6 ± 5.59	56.6 ± 2.70	44.1 ± 5.44
MSP	Inlet	34.9 ± 0.73	76.7 ± 2.27	73.5 ± 2.90	78.9 ± 2.49	53.8 ± 1.4
	Effluent	52.0 ± 1.82	73.5 ± 3.43	68.7 ± 1.644	69.8 ± 1.37	62.7 ± 0.25

**FIGURE 2:**
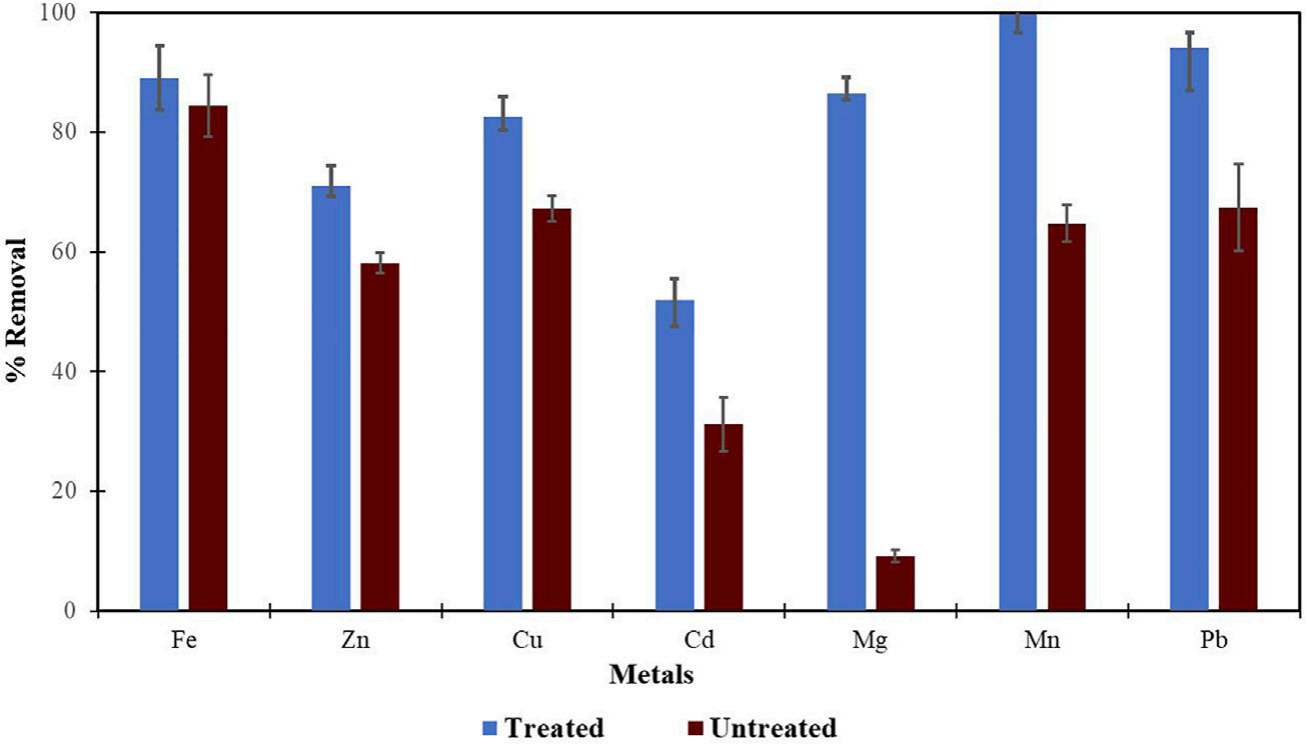
Comparison of acid treated and untreated MNS for removal of metals from wastewaters at inlet point.

**FIGURE 3:**
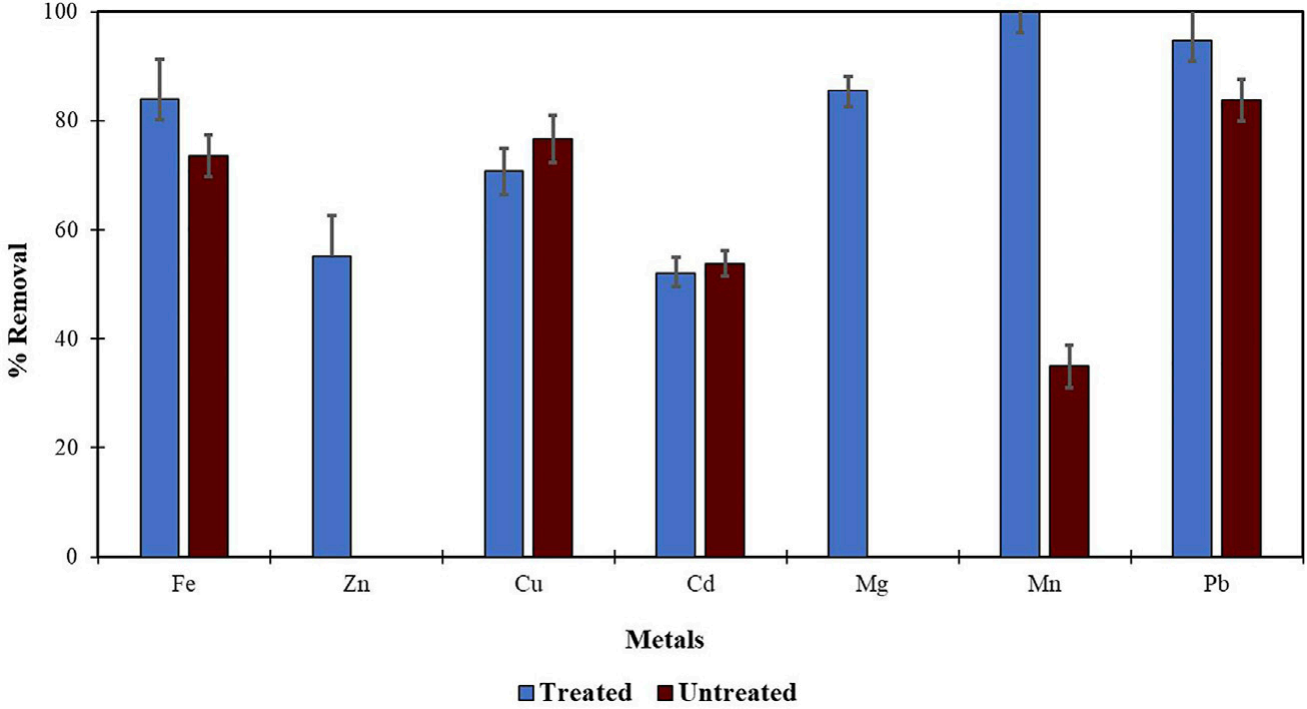
Comparison of acid treated and untreated MSP for removal of metal from wastewaters at inlet point.

Morula nutshells were found to be a better sorbent than Moringa seed pods as all metals showed a higher percentage of removal. Use of untreated plant biomass is capable of directly removing metal ionic species from aqueous solutions (Singh et al., 2006; Zwain et al., 2014). However, these sorbents can be chemically pre-treated to enhance better performance and/or suitability for process applications. This treatment normally removes organic and inorganic matter from the sorbent surface. Chemical treatments that are commonly employed are alkaline solutions, phosphoric acid, and citric acids. After acid treatment using 0.4 M of HNO_3_, the treated MNS and MSP sorbents were used for the extraction of heavy metals from the wastewater samples. To compare their effectiveness, the treated MNS and MSP were used in effluent wastewater samples. There was increased removal efficiency for all the metals, as shown in Figures 2, 3. There was an increase in removal efficiency. Zn ions were desorbing before the sorbents were treated, but it was noticed that the removal increased up to 55.6% for Zn. The increased removal efficiency could be because more binding sites were available after treating the sorbents, and hence more ions could bind to many adsorption sites.

**FIGURE 4:**
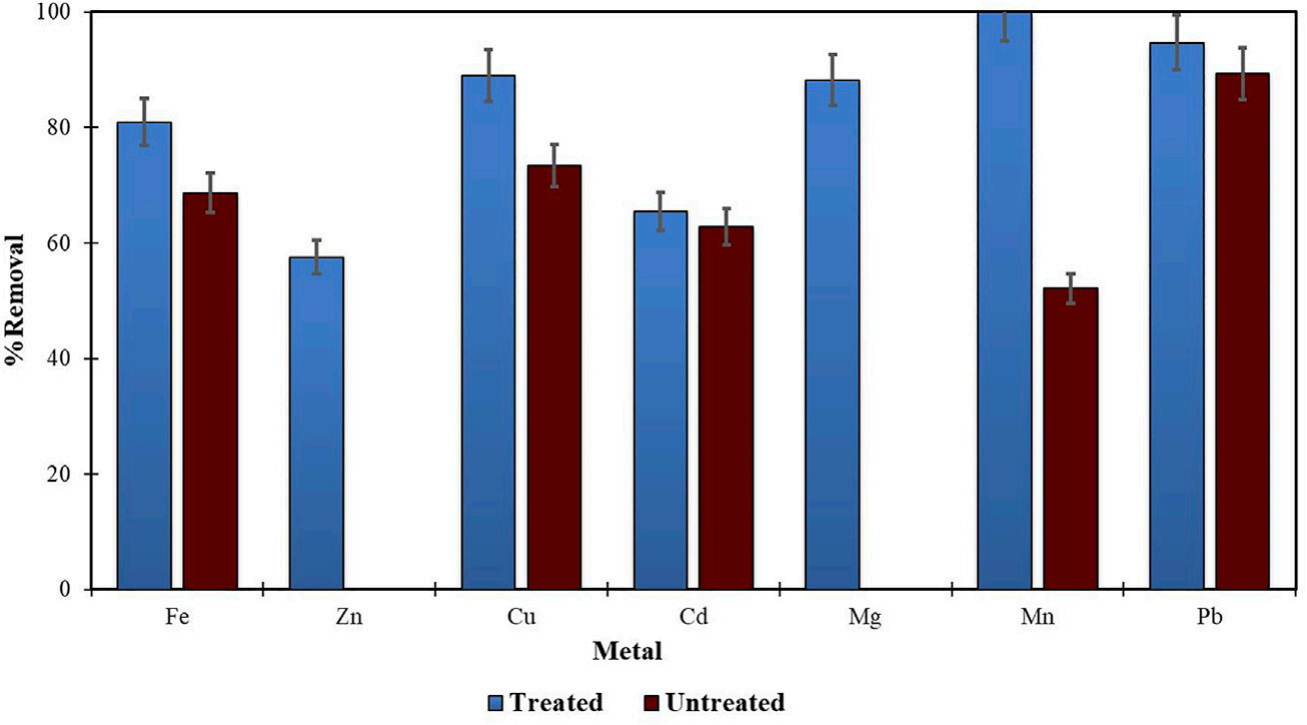
Comparison of treated and untreated MSP for removal of metal from wastewaters at final effluent point.

Treating the sorbents with acid helps to extract any metal cations on the sorbents, thereby creating new sorption sites and increasing the surface area of the sorbent and hence increasing the metal removal efficiency (Chan, et al., 2006; Gupta and Babu 2009). Acid-treated sorbents like maize bran (Singh et al., 2006) and tea waste (Mahvi et al., 2005) have been reported to have a high metal removal efficiency of >90%.

Conditions such as contact time, pH, temperature, particle size, sorbent dose, and initial metal concentration were optimized for MSP and MNS for the removal of selected metal ions (lead, cadmium, copper, manganese, iron, zinc, and magnesium) from wastewater and borehole water samples (Maina et al., 2016). The acid-treated sorbents showed higher removal efficiencies than the non-treated sorbents. These non-edible plant parts of Morula and Moringa plants are proposed as a cheap, simple, and effective alternative for the purification of water contaminated with heavy metals.

Metal ions such as potassium, calcium, and magnesium were found to be in higher concentrations in Moringa seed pods, implying that the pods can be a source of these essential elements. Morula nutshells had relatively high concentrations of sodium, potassium, magnesium, and aluminum. Other metals such as zinc, copper, iron, lead, and manganese were found to be at trace levels. All selected metals were within the limits set by FAO/WHO (2017).

The wastewater collected from the GVWTP was found to be conducive for agricultural use. On the other hand, the water from the boreholes was found to be good for human consumption. The concentration of the selected metal ions in both water samples was within the required limits for agricultural uses (wastewater) and drinking water (borehole) set by the US EPA. Method recoveries were obtained in the range of 86.49 ± 1.53% to 99.63 ± 1.99%. The low detection limits were also recorded (lower than 2 ppm) with a good linear correlation (*R* ^2^ > 0.99). Removal of the selected metals from the water samples by the sorbents could be through adsorption, ion exchange, or through precipitation where the sorbent would act like a filter.

Heavy metals were detected in the borehole water (Table 7) albeit at very low concentrations, which are below the maximum allowed limits (MALs) suggested by WHO (2017). This could be because the farm is very far from the urban environment and is therefore not prone to pollution from the industrialization activities, which are normally carried out in urban places.

**TABLE 7:** Heavy metal content of borehole water.

Metal	Concentration (mg/L)	RSD (%)	MAL* (mg/L)
Fe	0.3440 ± 0.0036	1.04	None
Zn	0.0110 ± 0.0021	19.09	None
Cu	0.0123 ± 0.0012	9.75	2.0
Cd	0.0011 ± 0.0001	10.01	0.003
Pb	0.1133 ± 0.0321	28.33	0.01
Mn	0.0012 ± 0.0009	75.02	None

* Maximum allowed limits (WHO, 2017).

Only lead occurred at levels higher than its MAL (0.01 mg/L). This could be because the farm is near the road, where fumes from motor vehicles could have an impact on the soil and subsequently the groundwater below that soil. The presence of iron, manganese, and magnesium in the water is beneficial to both farm animals and humans.

## 4 Discussion

### 4.1 Nutrient analysis

Agricultural wastes such as maize stalks, moringa seed pods, and Jugo beans husks were found to contain NO_3_^−^, NO_2_^−^, and PO_4_^3-^ in considerable amounts. Recycling of these agricultural waste materials is economic and eco-friendly due to their unique chemical composition, availability in abundance, renewability, and low cost. In addition, these substances contain organic matter which improves the soil’s structure, water-holding capacity, water infiltration, aeration, pH, and buffering capacity.

The farm from which the borehole water was obtained has no supply of potable water, and its inhabitants (both humans and animals) depend largely on borehole water. This is a common situation in Botswana as farms are very far from municipal areas, which are supplied with treated, potable water and proper sanitation measures. Due to this, the inhabitants of farms normally depend on private borehole water supplies, which are drawn from groundwater of uncertain quality.

The presence of NO_3_^−^ and NO_2_^−^ in drinking water is a concern because the microbial reduction process in food products and saliva can convert NO_3_^−^ to NO_2_^−^ (Burakham et al., 2004). NO_2_^−^ is harmful to human health primarily because of its ability to react with the ferrous ion (Fe^2+^) in the blood’s hemoglobin to produce methemoglobin (Jobgen et al., 2007; Bellavia et al., 2013). Normal hemoglobin in the blood contains iron (II). Nitrite can oxidize this iron to the iron (III) state, resulting in a molecule that has no oxygen-carrying capacity. This condition limits the blood’s ability to carry oxygen from the lungs to the rest of the body, giving rise to a situation called methemoglobinemia, which might be described as chemical suffocation (Jobgen et al., 2007; Makridin et al., 2020). According to Cemek et al. (2007), it has been ascertained that in severe cases of methemoglobinemia, the deprivation of hemoglobin and oxygen delivery to the brain may lead to intellectual disability.

Groundwater phosphate occurs naturally from the weathering processes of rocks and from anthropogenic activities. Investigations of farming practices need to be undertaken to ascertain the trend of phosphorus in water, plants, and soil on this farm.

### 4.2 Metal analysis

The Glen Valley Wastewater Treatment Plant (GVWTP) in Gaborone, Botswana, uses the activated sludge process (ASP) as the main technique of treating wastewater. This is a biological treatment system in which a specific concentration of biomass is maintained along with a sufficient dissolved oxygen concentration in an aeration tank that serves as a bioreactor—the purpose of which is to enhance the effect of biodegradation of soluble organic impurities (Mittal, 2011). The main products of this process in wastewater are sludge, water, carbon dioxide, and methane. Heavy metals also occur at concentrations that are quite higher than their toxicity limits and strongly affect and reduce microbial activity. This, in turn, has a negative impact on the biological wastewater treatment processes, and it leads to the reduction of the microbial oxidation of organic compounds and inhibition of the nitrification and denitrification processes (Kapoor et al., 2015). It is for this reason that many industries which produce effluents which are heavily contaminated with heavy metals are investing on on-site effluent treatment facilities to reduce the concentration of heavy metals (Ahluwalia and Goyal, 2007; Kurniawan et al., 2006).

The major source of wastewater in sewage treatment plants is municipal wastewater which may contain pathogens and potentially toxic elements and organic compounds. If ingested, these substances may result in health issues such as diarrhea. Diarrhea is responsible for the deaths of 1.8 million people every year, and it mostly affects children in developing countries because of unsafe water supplies and poor sanitation facilities. It is also of critical concern that additional traces of inorganic compounds such as heavy metals could be present in treated water due to treatment failure or inability to remove them (Qureshi et al., 2016). Wastewater resulting from homes and industries is often discharged into rivers after treatment. In studies conducted by Okuda et al. (2001) and Ghebremichael et al. (2005), the presence of bivalent cations such as Ca^2+^ and Mg^2+^ significantly enhanced the coagulating properties of MO seeds, where the cations may have adsorbed to the active components to form an insoluble net-like structure to capture suspended particles of kaolin clay that was present in water samples. The active ingredients extracted from MO seeds are cationic and able to remove dirt in water which is normally negatively charged (Kwaambwa and Rennie, 2012; Moulin et al., 2019). MO seed biomass were used to remove several metal ions including Pb, Cu, Cd, Ni, Mn, and Zn (Sharma et al., 2006; Obuseng et al., 2012). An attempt to add value to waste material by developing an eco-friendly method for removing Pb, Cu, Cd, Zn, and Fe ions from aqueous water was conducted (Maina et al., 2016). These studies demonstrated that pH, particle size, sorbent dose, contact time, temperature, and concentration are significant factors in adsorption. The percentage removal efficiency of Fe, Cu, Cd, Zn, and Pb ions using treated *Moringa oleifera* seed pods (MOSP) was found to be 99.0 ± 4.27, 98.7 ± 3.89, 72.5 ± 2.14, 65.9 ± 1.64, and 99.6 ± 6.69, respectively. This work also demonstrated that the performance of this sorbent could work at a wide range of pH 3.5 to 8 and that it was possible to adsorb a mixture of metal ions without significantly affecting sorption capacity. At a high pH, the surface charge of the sorbent is negatively charged and therefore the sorption of metal ions on the sorbent is high. At a low pH, the metals ions compete with the H^+^ ions for the active sites, therefore decreasing the sorption (Guyo et al., 2015). In the study of Bhatti et al. (2007), biomass from MO pods was also used to investigate the removal of Zn(II). It was compared to the biomass treated with NaOH and noted that removal efficiency increased from 36.07% to 45.76% when treated biomass was used. The experimental results showed that the maximum pH (pH max) for efficient sorption of Zn(II) was 7 ± 0.1 at which evaluated biosorbent dosage and biosorbent particle size were 0.5 g/L and <0.255 mm, respectively. An increase in sorbent dose increases the number of particles and hence increasing the removal efficiency due to an increase in number of the binding sites for sorption. Small particle size also increases the sorption capacity as results of larger surface area for the adsorption of the metal ions onto the sorbent (Arunlertaree et al., 2007). The seed pods have also been used to remove organics (benzene, toluene, and cumene) from aqueous solution (Adeleye et al., 2016). It was observed that the removal of the organic contaminants onto the pods was in the order of cumene > toluene > benzene. This was attributed to their solubility, as more soluble substances in alcohol and water prefer to remain in solution rather than the sorbent material. However, some parts of these plants are not edible and are considered waste, for example, MO seed pods and Morula nutshells. MO bark was considered a viable alternative to activated carbon, ion-exchange-resin, and other synthetic adsorbents used for this purpose (Reck et al., 2018; Viotti et al., 2019). The use of this plant waste material demonstrates effective, economic, and available methods that can be used to remove metal ions from water and wastewater and therefore applicable even in rural areas where resources to acquire the expensive and complicated technologies are limited. Minimal studies have been directed toward sorption behavior for the removal of toxic metals from water samples using the nonedible plant parts of MO and Morula trees.

Recycling and reuse of treated wastewater can be a supplementary source to already existing water sources, especially in arid and semi-arid regions.

Nonedible plant parts of *Moringa oleifera* seed pods and Morula nutshells were used to remove selected heavy metals from water samples. The biosorption capacity of agricultural waste has also been demonstrated, hence revealing the potential of the use of agricultural waste for the purification of water. This introduces possibilities of recycling and reuse of water generated by industries and wastewater treatment plants, etc., to curb the problem of water scarcity. The developed method can be used in rural areas where there are no resources to obtain the conventional expensive techniques. To improve efficiency, both Moringa seed pods and Morula nutshells were acid-treated with nitric acid (HNO_3_), and the percentage removal was increased due to an increase in surface area that ultimately increased the binding sites of the sorbents. Although the concentrations of the selected metal ions in the water samples collected from the Glen Valley Wastewater Treatment Plant were found to be within the required limits for agricultural uses set by the US EPA, they were much higher than the required limits for the water to be used for drinking purposes. Optimization of pH, initial metal concentration, sorbent dose, particle size, and temperature helped to achieve desirable extraction efficiencies.

## Conclusion

The study demonstrated a simple, cheap, and environmentally friendly method of remediating wastewater without the need for trained personnel. It emphasized on the importance of utilizing all unwanted biomass materials such as agricultural waste for reclamation of wastewater. The method can be used in rural areas where there are no resources to obtain the conventional expensive techniques and chemicals. In addition, this is a demonstration of green chemistry and its applicability in the African continent, which quite often does not afford expensive fertilizers and water purification systems from developed economies. The use of agricultural waste also adds value to the plants from which the waste has been obtained.

## Data Availability

The original contributions presented in the study are included in the article/Supplementary Material; further inquiries can be directed to the corresponding author.
